# Ethical Issues in Democratizing Digital Phenotypes and Machine Learning in the Next Generation of Digital Health Technologies

**DOI:** 10.1007/s13347-021-00445-8

**Published:** 2021-03-21

**Authors:** Maurice D. Mulvenna, Raymond Bond, Jack Delaney, Fatema Mustansir Dawoodbhoy, Jennifer Boger, Courtney Potts, Robin Turkington

**Affiliations:** 1 School of Computing, Ulster University, Shore Road, Newtownabbey, Northern Ireland UK; 2 Imperial College School of Medicine, Imperial College London, South Kensington, London, UK; 3 Department of Systems Design Engineering, University of Waterloo, University Avenue West, Waterloo, Canada

**Keywords:** Ethics, Digital health, Ecological momentary assessment, Experience sampling method, Unsupervised machine learning, Digital phenotyping, Event log analysis

## Abstract

Digital phenotyping is the term given to the capturing and use of user log data from health and wellbeing technologies used in apps and cloud-based services. This paper explores ethical issues in making use of digital phenotype data in the arena of digital health interventions. Products and services based on digital wellbeing technologies typically include mobile device apps as well as browser-based apps to a lesser extent, and can include telephony-based services, text-based chatbots, and voice-activated chatbots. Many of these digital products and services are simultaneously available across many channels in order to maximize availability for users. Digital wellbeing technologies offer useful methods for real-time data capture of the interactions of users with the products and services. It is possible to design what data are recorded, how and where it may be stored, and, crucially, how it can be analyzed to reveal individual or collective usage patterns. The paper also examines digital phenotyping workflows, before enumerating the ethical concerns pertaining to different types of digital phenotype data, highlighting ethical considerations for collection, storage, and use of the data. A case study of a digital health app is used to illustrate the ethical issues. The case study explores the issues from a perspective of data prospecting and subsequent machine learning. The ethical use of machine learning and artificial intelligence on digital phenotype data and the broader issues in democratizing machine learning and artificial intelligence for digital phenotype data are then explored in detail.

## Introduction

The main focus of previous research on the analysis of data for digital wellbeing technologies used in health and wellbeing has been to aid in usability analysis, user adoption/retention analysis (Miller et al., 2007), or to reveal usage patterns in using technology (de Santana & Baranauskas, 2010). Research has also been carried out to explore how rehabilitation devices can have data or event logging incorporated, but this has been more to support the goal of device monitoring (Woo & Mori, 2004). More recent research has examined engagement data in web-based intervention platforms but has primarily focused on the visualization of user log or user event data (Morrison & Doherty, 2014).

Digital phenotyping is the term given to the capturing and use of user log data from health and wellbeing technologies used in apps and cloud-based services (Insel, 2018; Martinez-Martin et al., 2018; Torous et al., 2018). Digital phenotyping was originally proposed as a way to correlate a person’s mental state by using their metadata and even sensor data on their smartphone. In some cases, the data is physiological, for example, pulse or movement-related, and it is collected automatically. In other cases, the data is actually metadata, for example, when a call is made and the call duration rather than the content of the call (O’Neill et al., 2019). Oftentimes, as would be expected from a personal device located on the body of the user, rich data pertaining to geo-location, social media use, and interaction is gathered. Health and wellbeing-related, scientifically validated assessment scales may also generate digital phenotype data. Another form of digital phenotype data is the experience sampling method (ESM) (or the ecological momentary assessment or EMA) (Lewin, 1935), which originally made use of paper-diary techniques to enable people to record their observations or answers to specific questions and combined the ecological validity with the rigorous measurement techniques of psychometric research. EMA secures data about both behavioral and intrapsychic aspects of individuals’ daily activities, and it obtains reports about the experience as it occurs, thereby minimizing the effects of reliance on memory and reconstruction which can often be impaired by hindsight bias or recall bias (Reed & Csikszentmihalyi, 2014). The use of digital phenotyping data and its analysis using machine learning and artificial intelligence is important since many national public health organizations including the UK’s National Health Institute (NHS) are exploring how to use digital technologies such as health apps and cloud-based services for the self-management of diseases, and thus logging user interactions allows for greater insight into user needs and provides ideas for improving these digital interventions, for example, through enhanced personalization. Public health services benefit since the data can be automatically and hence cost-effectively collected. Such data may facilitate new ways for digital epidemiological analyses and provide data to inform health policies. If the public health organizations promote health apps and digital phenotyping analysis using machine learning and artificial intelligence is taken up by these organizations, then there is clear need for guidelines on the ethical application of these “democratized” algorithms and techniques.

## Digital Phenotyping Workflow

Standardizing workflows is crucial in order to ensure consistency and that best practices are adopted in a domain. A number of standard workflows for using machine learning and artificial intelligence have been proposed. For example, the cross-industry standard process for data mining (CRISP-DM) is a data mining process model encompassing the following stages: business understanding, data understanding, data preparation, modeling, evaluation, deployment (Shearer, 2000). CRISP-DM has been available in various guides since 1996. An updated variant developed by IBM, called Analytics Solutions Unified Method for Data Mining/Predictive Analytics (ASUM-DM), expands on CRISP-DM (Haffar, 2015). The workflow for Health Interaction Log Data Analysis (HILDA) involves three high-level phases of data preparation, data prospecting, and machine learning (Mulvenna et al., 2018).

The acquisition and preparation of data (behavioral data, social media data, metadata, EMA data) is the first stage in the digital phenotyping workflow. The second stage is data prospecting, where the data are examined to identify how best to apply machine learning and artificial intelligence techniques. The third stage is the application of machine learning and artificial intelligence techniques to discover actionable and useful insights from the data. Stages 2 and 3 are where significant knowledge is required in order to understand which techniques should be used and why. The fourth stage of the workflow is to provision the insights on service delivery platforms so that they can be made use of in the apps and cloud-based services, either directly to the user as personalized service recommendation for example, or to the service provider as aggregated user insights, for example, including real-world data (Mahajan, 2015).

## Issues in Democratizing Digital Phenotype Data

Democratizing digital phenotype data opens up both passive (sensor collected, for example) and active (EMA collected, for example) data for use. Table 1 outlines the main ethical concerns arising from collection of the different types of data.

**Table 1:** Ethical concerns on sensor data used in digital phenotyping (adapted from Rooksby et al., 2019)

Sensor	Description	Area of concern	Ethical concerns
Accelerometer	Acceleration of moving person or device	Activeness	Concerns in this area stem firstly from the inference of a person’s activeness, which can be mitigated as long as confidentiality is maintained. However, determining an anonymized users’ home location from GPS and accelerometer data has been shown to be successful in 79% of users, with additional accurate behavior prediction using this data (Fuller et al., 2017)
App usage	Installation of apps, duration spent on apps, frequency of use	App usage	Here, the main ethical concern is focused on behavior analysis and prediction. The potential for the abuse of collected app usage data in third-party advertising is huge. Mohr et al. (2020) explained how 80% of the top 30 mental health and smoking apps shared user data for advertising, but only 28% declared this
Browser history	Previous websites visited	Online behavior tracked	While similar concerns around advertising are raised here, there is an additional concern around privacy. While users may consent to browser history tracking on a particular device, they may not be aware of the syncing of browser history across devices, and that they may be unknowingly agreeing to larger amounts of data sharing than they realize (Fisher et al., 2020)
Bluetooth	Nearby devices and status	Interconnectedness	In light of COVID-19 contact tracing, the main concerns raised here are focused on the lines being blurred between disease monitoring and population monitoring, with corresponding encroachment on human rights (World Health Organization, 2020). Additionally, looking at nearby users’ device data should not be possible without informed consent from those device users. It raises the question of how useful the data will be if you can’t consent everyone; therefore, is it ethically worth collecting
Call logs	Incoming and outgoing calls	Contacts and network analysis	Network analysis and call tracing allow for GPS/location data collection, which raises the same concerns as previously mentioned. Further analysis of contacts raises consent and privacy of gathering unconsented individuals’ data as discussed above
Camera	Detecting raw images, counting photos taken, image geotagging	Personal imagery	Obvious ethical issues around data privacy and storage of photographs and their data. Particularly in healthcare settings, when photographs may constitute sensitive data for GDPR purposes if they contain information that relates to mental or physical health
Screen	Status of screen, on or off	Attraction to device	Studies have shown that screen activity can be harnessed for predicting behavior and personality types (Stachl et al., 2020). Such data could be used by third-party organizations for targeted advertising so confidentiality and privacy is paramount
Keyboard/UI	Event counts, potentially recording keystrokes	Interaction with device	This might not seem like an ethical concern like the others; however, this is a form of passive data collection. Security and storage of this form of data is extremely important and a major ethical concern for passive data. Worries circulate around the distribution or sale of the data collected. (Senders et al., 2019)
Location	Geographical coordinates of device	Location tracking	Location tracking is a major concern of the twenty-first century. It raises concerns about privacy and confidentiality. It is also challenging to ensure all users are appropriately informed about the implications of GPS tracking. Another danger of location tracking is the unauthorized use of the data by third-party organizations. (Apte et al., 2019). Additionally, concerns have been raised around “in situ” data collection, when a user is at home, and the extent of how much a user remembers or realizes that they are being monitored (Maher et al., 2019)
Microphone	Recording of sound including decibel level	Audio surveillance	A key ethical concern about audio surveillance is privacy. Enabling microphone on smart devices can allow recordings of private conversations, collecting passive data without consent. Data protection is critical in digital phenotyping due to the sensitivity of health records and information. (Martinez-Martin et al., 2018)
SMS/email	Messages sent and received	Content analysis	Along with privacy and confidentiality issues, there is another issue of transparency. Users need to be told what data is collected, how it is collected, and when it is collected. The app needs to clearly lay out to users what data is and is not collected, including external apps such as SMS and email (Martinez-Martin et al., 2018)

Digital phenotype data, while open to facilitation by users, is also then more susceptible to the usual issues around data. It can be stolen, used, or analyzed for criminal purposes.

## Case Study—Reminiscence Health App User Log Data

This case study reports on the analysis of log data from a tablet application, specifically designed and developed to facilitate reminiscence for people with early- to moderate-stage dementia. Reminiscence is the sharing of memories relating to personal life experiences. It is the act of remembering and reflecting on real past events. The act of reminiscing can serve many functions that create bonds between people and, in doing so, support them to reflect on important life events and to attribute meaning to their lives (Butler, 1963). The development of the app was a component part of a larger feasibility study to investigate the effects of individual specific reminiscence activity using a range of outcome measures, to explore users’ views on the app, and to incorporate an economic analysis, examining the cost of implementing the app intervention in comparison with quality of life outcomes. The feasibility study incorporated a paired sample of 28 dyads (person living with dementia and their carer) and applied several scales at start, mid, and end point of a 12-week use of the app in the homes of people living with dementia and their carers, with one-to-one interviews with participants carried out at the end of the 12 weeks.

## Data Prospecting

The app was designed to incorporate a logging facility for key events by users across 45 specific activities, covering five different types of events. The five different canonical events include entry (logging in), admin (adding a photo, deleting an audio, etc.), reminiscing (viewing a video, viewing a photo, etc.), in the moment (ITM) questions, and exit (logging out). Thus, the behavior of users can be analyzed within and across each usage session, over the 12-week trial. The ITM questions comprise items from the primary outcome measure for the study, the Mutuality Scale developed by Archbold et al. (1990).

The data show that the app was primarily used for reminiscing as expected. A total of 71% of interactions from people living with dementia were within the reminiscing sections of the system whereas only 47% of interactions from carers were within the reminiscing sections (*p* < 0.001). It is reassuring that people living with dementia mainly used the system for reminiscing. Only carers could carry out “Admin” events such as adding a photo, as mandated by their access rights set at login. It can perhaps be seen as a positive sign that carers generally added to the music, pictures, and videos that were uploaded to the app prior to the intervention beginning, rather than simply browsing those already there. There were twice as many interactions with photographs in comparison to music and five times as many interactions with photographs in comparison to video by people with dementia using the app. Reminiscing, with its history in photograph-based memory books, has been more about the image than music, sound, or video, and this effect may be what is being seen in this data (Wright, 2009). What is also interesting in this data is the popularity of music to people living with dementia. Again, this is known from the literature (Sixsmith & Gibson, 2007) and anecdotally from carers of people living with dementia but it is useful to see this behavior replicated in this trial data. The most popular times that the dyads of people living with dementia and carers prefer to use the app peak around 11am, 3 pm, and 8 pm. These times correspond to post-breakfast, post-lunch, and post-evening mealtimes. The number of unique days in which users interacted with the system was calculated, and there is a significant statistical correlation between the number of days the carer interacted with the system and the number of days the dyad’s corresponding person living with dementia interacted (*r* = 0.577, *p* < 0.001).

## Machine Learning

In this study, K-means clustering algorithm was used, given it is the most widely used and established clustering algorithm in the unsupervised machine learning literature. Using the elbow method, 4 was discerned as a reasonably small number of clusters that would provide reasonable resolution in terms of explained variability. Clustering was based on the following five features: number of interactions by person living with dementia, number of interactions by carer of person living with dementia, number of daily interactions by person living with dementia, the mean usage interval by a user, and the standard deviation of usage interval by a user.

Four clusters were revealed by the K-means algorithm. The first cluster, “the hooked adopter,” constituted one dyad, who fully adopted the system. They had 7.2 times more interactions than their carer. While the person with dementia used the app with high frequency, the carer showed a normal amount of usage; hence, the person with dementia was independently dedicated. The “hooked adopter” dyad uses the app for over half the days in a month (55% of days) and with little variability uses the app every 2 days. The second cluster, labelled the “typical user,” encompassed the plurality of users, where 12 dyads or 43% fall into this cluster, hence making them the most typical user. These people living with dementia user only have 1.7 times more interactions with the app than their carer. This indicates that these users have some dependence on the carer for app usage. This dyad uses the app 15% of days in a month. This dyad is unpredictable when they will use the app but on average interacts with it every 6.61 days (approximately once per week). The third cluster, labelled “disengaged irregular user,” encompassed 7 dyads or 25% of users. These users had 25% fewer interactions with the app than the carer. While the people with dementia had fewer interactions than their carers, the carers had fewer interactions than other carers in all other clusters. These dyads use the app 9% of the days in a month. However, typically they can go for 20 days without using the app making them the least consistent users of the app. The final cluster, labelled the “well-supported dependent user,” encompassed 8 dyads or 29% of users, the second largest group of users. These users have 36% fewer interactions with the app than their carers. The carers are very enthusiastic and have more interactions than other carers in all other clusters but they seem to struggle to get people with dementia users to the same engagement level. Similar to the typical users in cluster 2, these dyads interact with app 16% of the days in a month and on average use the app every 6.97 days. This unsupervised learning provided clusters that were clear and transparent to the health science researcher involved in the project. The next stage in this work is to seek to identify correlations between the post-trial interviews with the dyads and the clusters enumerated above.

## Ethical Use of Machine Learning and Artificial Intelligence on Digital Phenotype Data

The four ethical pillars of medicine are autonomy (right to choice), beneficence (doing good), non-maleficence (do no harm), and justice (equal access), and these pillars should not be overlooked when democratizing digital phenotyping. The entire workflow for the use of digital phenotyping data raises significant ethical concerns, covering accountability, protection of user data, transparency, and informed consent (Martinez-Martin et al., 2018). Intended use and informed consent cover autonomy as patients need to be aware of how the app and digital phenotyping will be used before consenting to the T&Cs. Explicit and unambiguous language is crucial and must make clear intended use T&Cs in digital phenotyping to ensure accurate, informed consent is made (Dagum & Montag, 2019). Nowadays, most users maneuver the T&Cs carelessly and haphazardly due to its complex and dense nature. This raises the concern that users have not given proper informed consent. In medical settings, it is imperative to explicitly define to patients how their data is collected, stored, and used regarding their medical care. Incorporating digital phenotyping into a patient’s EHR (electronic health record) introduces a new concern of potentially unconsented third-party access to the EHR. Understanding human nature and the ethical pitfall it opens, steps need to be taken to improve technology’s consent processes. Key information should be signposted and highlighted to ensure user acknowledgment. GDPR has already laid out clear guidelines on how consumers must be informed concisely and in plain, simple language how their data is collected and processed (Martinez-Martin et al., 2018).

There are specific concerns relating to the second and third stages, with the ongoing “democratization” of machine learning and artificial intelligence in this workflow (Bond et al., 2019). While democratizing machine learning and artificial intelligence by making them more accessible can be a force for good, it is essential to consider potential negative ramifications. For example, there are ethical implications since such usable machine learning and artificial intelligence tools could increase inadvertent unethical use cases of artificial intelligence due to ignorance and lack of machine learning and artificial intelligence literacy amongst their lay users. One could argue that usable machine learning and artificial intelligence are analogous to allowing people to drive cars without any knowledge of car mechanics. And while this is the case, drivers do need to know “how” to drive a car and understand the hazards of driving. Likewise, usable machine learning and artificial intelligence should be complemented by some machine learning literacy—a form of general literacy in machine learning and artificial intelligence bearing in mind the risks of machine learning and artificial intelligence deployment. There are many examples of the unethical use of machine learning and artificial intelligence, including the use of machine learning and artificial intelligence to predict sexuality, the use of facial detection software that only works with certain demographics, and the use of judicial machine learning and artificial intelligence systems that over-predict reoffending rates amongst certain groups. It is interesting to note that other data scientists have picked up on the potential unfairness of applying big data in the next generation of data-based products and services (O’Neill, 2016). A significant twenty-first-century example of this is the Amazon AI recruitment scheme. The Amazon AI software was introduced to reduce the human bias, but as it was taught with a biased dataset, it too regarded male resumes as more preferable than females. Even though their program was edited to maintain neutrality, there is no guarantee that machine learning or artificial intelligence will not lead to concerns around discrimination (Dastin, 2018).

As technologies become increasingly complex, pervasive, and interconnected across different disciplines, some call for more ethically sound underpinnings for product and service technology development (Mulvenna et al., 2017). For example, it can be seen that those in the machine learning and artificial intelligence community recognize that the context and positioning of next-generation intelligent systems that will likely monitor people or impact in their lives in unknown ways need to be explored and researched by calling for “Fairness, Accountability, and Transparency” (FAT/ML, 2016). For example, it is important to consider the “data provenance” of a dataset that is used in machine learning. Data provenance comprises the history of the dataset, where and how it was collected along with all its potential biases and nuances. Using a machine learning model in the real world to make decisions could be considered unethical if the data scientist did not consider overfitting to noise in the dataset or if some features in the model could be considered as “data leakage” or indeed the notion that a machine learning model has a shelf-life due to “concept drift.” Ignorance of such phenomena is unethical and would result in misrepresented and unrealistic promises of any results that are produced.

Digital phenotyping can help users on an individual level and on a larger population level as all the data collected can provide invaluable insight into disease progression and development on a global scale. This area is where the boundaries blur and ethical issues emerge. Digital phenotyping can help the masses and the individual, but using digital phenotyping for public health research purposes needs informed consent and transparency with the users as it does not directly benefit the user contributing their data. In a standard clinical setting, in order to use patient data in secondary situations, the patients need to be reconsented before using their data again. However, it is growing difficult to set solid boundaries regarding data access with technology and, in particular, digital phenotyping (Martinez-Martin et al., 2018).

Digital phenotyping can also manifest into a spin-off condition called cyber-hypochondria (a compulsion of constantly and obsessively monitoring one’s own digital health data due to an anxiety of falling ill, an example of de-corporealization) (Stanghellini & Leoni, 2020). This raises the question: are we harming the patient more than we are helping them? As the users do not have the same medical teaching as health professionals, they do not have the ability to distinguish normal reading from abnormal in a dichotomic way. This can produce obsessive natures in patients, constantly monitoring themselves and their digital health, anxiously studying any variation in their trends due to worries it may indicate some underlying health problem. As much as we are trying to help the users, we are also in turn hurting them unknowingly. Furthermore, research into health anxiety during the COVID-19 pandemic highlighted how patients can be biased towards results, symptoms, and readings that point to a diagnosis (Cannito et al., 2020).

The potential of digital phenotyping is remarkable, and its impact on healthcare is vast. However, as the information revolving around digital phenotyping takes a sensitive and personal nature, many ethical concerns surround it. As with anything in the twenty-first century, digital phenotyping is rapidly evolving, which further pushes the ethical boundaries in the realm of machine learning and artificial intelligence. Robust ethical frameworks need to be drafted to ensure that patients and their information are protected in accordance with the four ethical pillars while allowing digital phenotyping to provide the healthcare sector with the numerous potential benefits it has in the clinical, scientific, and public health fields.

## Issues in Democratizing Machine Learning and Artificial Intelligence for Digital Phenotype Data

There are many issues in democratizing machine learning and artificial intelligence for digital phenotype data, or indeed when analyzing any type of data from any source. In order to highlight the need for caution when working with digital phenotype data, these issues are identified and described in this section. They include data provenance and confounding; model selection and the “no free lunch” theorem; algorithm bias and fairness; model performance; prediction errors; responsibility; and automation bias.

Data provenance specifies trust in the source and location of data used to build the machine learning and artificial intelligence model (Glavic, 2014). Having reliable, good-quality data before applying machine learning and artificial intelligence modelling is essential as algorithms are only as good as the data they are trained on. Sampling bias is one issue which can affect the reliability of data, that is bias introduced during systematic data collection which can cause certain subgroups to be under- or over-represented affecting model performance. This includes, for example, selection or regional bias, where individuals are not chosen at random but instead selected based on their demographic or location. Additionally, a confounding variable, which may be a feature or predictor causing a spurious association with the outcome variable, can result in machine learning and artificial intelligence algorithms under or overestimating in a model.

Supervised machine learning involves training an algorithm to learn patterns from data which allows prediction or classification of an outcome when given unseen cases. There are many techniques that can be used in machine learning and artificial intelligence (Domingos, 2015). The issue is that no one algorithm solves all problems across all disciplines, known as the “no free lunch theorem” (Wolpert & Macready, 1997). As there are very large amounts of approaches available, it is important to establish the optimal machine learning and artificial intelligence technique depending on the problem. To ensure the user does not rely on one method to solve all problems, it is critical to assess the strengths, weaknesses, and assumptions of the different algorithms and have basic awareness of how the technique was developed.

Algorithmic bias is when a machine learning and artificial intelligence model discriminates, for example, against race or gender (Hajian et al., 2016). It is important that the chosen algorithm is fair. Fairness in the sense of machine learning and artificial intelligence can be thought of as algorithms that do not discriminate based on an individual’s protected class status, for example race, sex, or religion (Friedler et al., 2019). Careful consideration can be given to use fairness-aware algorithms that strive to adjust input data so the outcome or outputs will be fair by ethical standards.

A number of factors can affect the performance of a chosen model. Overfitting is one issue, where machine learning and artificial intelligence algorithms are modelled too closely on random noise within the training data and therefore do not perform well on unseen data.

A variety of metrics are available in machine learning and artificial intelligence models to assess performance. One such measure is accuracy, the fraction of correct predictions. It is important for the user to have an understanding of this when choosing the final model. Arguably, sensitivity (identification of true positives) and specificity (identification of true negatives) are the most important measures to compare different models. It is important to have a good overall understanding of these different metrics to select a model that is fit for purpose.

Predictors which are used in machine learning and artificial intelligence which appear indiscriminate may inadvertently have predictive capability. This is known as “data leakage,” where the solution is accidently used in training the model resulting in high performance (Kaufman et al., 2012). It is imperative that these “leaked” features are not used in the training stage as resulting algorithms may have low performance on real-world data.

Identification of type 1 errors (false positives) and type 2 errors (false negatives) is vital in developing a machine learning and artificial intelligence model so users can design their models to avoid them. Sensitivity or specificity can be used for algorithm selection depending on what the preference is for a particular sector or problem.

Responsibility in this sense refers to the individual who is accountable for launching the machine learning algorithm. This responsibility is down to the user, and careful consideration should be taken before the procedure is deployed. Automation bias can also occur when people rely on the results of an automated system despite the fact it may be producing incorrect results (Parasuraman & Manzey, 2010). It is important to make recipients aware of accuracy and limitations of the model and to avoid automation bias.

## Discussion

Digital phenotyping workflows can help ensure reproducibility of findings as knowledge is derived from digital phenotype data and they also support consistency and accuracy. The growth in data arising from the increased uptake and use of technology, apps, and cloud-based services relating to digital health, together with data growth from the democratization of machine learning and artificial intelligence techniques raises significant ethical issues when considering digital phenotype data. These are most pertinent to digital phenotype data in the first stage of the workflow, and in the second and third stages of the digital phenotype workflow when machine learning and artificial intelligence techniques and models are being considered for selection and use.

This paper has considered and discussed the most important issues that pertain to ethical use of machine learning and artificial intelligence approaches, which are data provenance and confounding; model selection and the “no free lunch” theorem; algorithm bias and fairness; model performance; prediction errors; responsibility; and automation bias.

Broader topics such as data protection and compliance with ethical guidelines also need to be accommodated within these types of workflows, especially as the EU General Data Protection Regulation (GDPR) (https://www.eugdpr.org/) came into force in 2018.

Digital phenotype data is being democratized as people elect to use apps and cloud-based services for self-management of their health. Machine learning and artificial intelligence are also being democratized as tools and techniques are being made available beyond the historical user base of specialized data scientists. Collectively, significant ethical questions arise across the entire digital phenotype workflow. Who benefits from this democratization? Is it beneficial to the users, the public health organizations, or both groups? Are there scenarios where the “greater good” outweighs the loss of personal autonomy? Can users elect to opt-in and opt-out, and what are the public health issues of users having and exercising these options? Should those undertaking machine learning of digital phenotype data require certification of the skills and knowledge of the process? The overarching ethical issue, therefore, lies in finding the balance between escalating new discoveries versus false discoveries via democratization of digital phenotyping data and machine learning.

There is therefore the need for guidelines on good research practice for the ethical use of digital phenotype data as well as the application of these “democratized” machine learning and artificial intelligence algorithms and techniques on the digital phenotype data. The incorporation of ethical guidelines into digital phenotype workflows is a significant implementation challenge for public health organizations worldwide. The failure to achieve consensus on best practice is a clear and present risk to public healthcare policy makers and public health organizations in countries dealing with governance, research, and implementation of such digital technologies for health.

## Data Availability

Not applicable.
